# Prescription Department

**Published:** 1898-03

**Authors:** 


FOR INFLUENZA.

℞. Quin. pulph..............gr. xlv.
Pulv. digitalis }
Pulv. scillæ } aa .....gr xv.
Ext. opii............... gr. v.
Ext. glycyrrhizæ...........q. s.
M. Ft. pil. No. XXX. Sig: Four pills daily.—Pepper.—*Medical News.*

TINNITUS AURIUM.

For the subjective noises following acute catarrhal inflammation of the middle ear, dilute hydrobromic acid is recommended, as:

℞. Dilute hydrobromic acid...oz. i.
Aromatic elixir.........oz. ii
M. Sig: A dessert spoonful, well diluted, two to three times daily.

FOR PHARYNGITIS SICCA.

℞. Ac. carbolici.............. dr. i.
Tr. iodi.................... *m* v.
Tr. aloes .................*m* vii.
Tr. opii..................gtt. x.
Glycerini....................oz. i.
M. Sig: By means of an atomizer apply to the pharynx several times daily.—*Medical News.*

ANOREXIA.

℞. Tincture of cinchona...........
Tincture of colombo...........
Tincture of gentian....aa 5 gm.
Tincture of rhubarb.......3 gm.
Tincture of nux vomica...2 gm.
M. Sig: Fifteen to twenty drops with each meal.—*Journal de Medicine de Paris.*

